# The use of radio-collars for monitoring wildlife diseases: a case study from Iberian ibex affected by *Sarcoptes scabiei* in Sierra Nevada, Spain

**DOI:** 10.1186/1756-3305-6-242

**Published:** 2013-08-22

**Authors:** Samer Alasaad, José E Granados, Paulino Fandos, Francisco-Javier Cano-Manuel, Ramón C Soriguer, Jesús M Pérez

**Affiliations:** 1 Estación Biológica de Doñana, Consejo Superior de Investigaciones Científicas (CSIC), Avda. AméricoVespucio s/n, 41092, Sevilla, Spain; 2 Espacio Natural de Sierra Nevada, Carretera Antigua de Sierra Nevada, km 7, E-18071, Pinos Genil, Granada, Spain; 3 Agencia de Medio Ambiente y del Agua, Consejería de Medio Ambiente, Sevillam, Spain; 4 Departamento de Biología Animal, Biología Vegetal y Ecología, Universidad de Jaén, Campus Las Lagunillas, s/n, E-23071, Jaén, Spain

**Keywords:** GPS-GSM-radio collars, *Capra pyrenaica*, Sarcoptic mange, Disease control

## Abstract

**Background:**

Wildlife radio tracking has gained popularity during the recent past. Ecologists and conservationists use radio-collars for different purposes: animal movement monitoring, home range, productivity, population estimation, behaviour, habitat use, survival, and predator-prey interaction, among others. The aim of our present study is to highlight the application of radio-collars for wildlife diseases monitoring. The spread of wildlife diseases and the efficacy of management actions for controlling them propose serious challenges for ecologists and conservationists, since it is difficult to re-capture (or simply observe) the same animal in pre-determined temporal interval, but such difficulty is overcome by the use of gps-gsm radio collars.

**Methods:**

In the present study we report, for the first time to our knowledge, the use of radio-collars in the monitoring of Iberian ibex affected by *Sarcoptes scabiei* in Sierra Nevada mountain range, Spain. Twenty-five moderate or slightly mangy animals were radio-collared between 2006 and 2013.

**Results:**

The radio-collars allowed us to confirm the presence of resistance to *S. scabiei* within Iberian ibex population. Twenty (80%) of the collared animals recovered totally from mange, while the disease progressed in the other five Iberian ibex (20% of the collared animals) and the animals died. The average estimated recovery time of the resistant animals was 245 ± 277 days, and the estimated average survival time of the non-resistant Iberian ibex was 121 ± 71 days. Non-resistant animals survived at least 100 days, while all of them died with less than 200 days. Sixty per cent of the resistant animals were recovered with less than 200 days.

**Conclusions:**

We report, for the first time, the successful use of radio collars for wildlife diseases monitoring using Iberian ibex/*S. scabiei* as a model. By using radio collars we documented that most of the *Sarcoptes*-infected Iberian ibex are resistant to this disease, and we estimated the average time for Iberian ibex recovering from mange infection and the average survival time of the non-resistant ones. We expect wider use of radio-collars for wild animals diseases monitoring, affected/not-affected animals interaction, and treatment efficacy, among others.

## Background

The development of radio-telemetry and its replacement, nowadays, by Global Positioning System (GPS)-based research techniques, has revolutionized wildlife research by gaining additional insight into the secretive lives of animal movements and ecology [1,2].

Detailed ecological and conservation questions were revealed by the application of this technological approach (Radio- telemetry and GPS collar technology) on wildlife research: 1-animal movement patterns on large scale (migration or dispersal) and smaller scale (spatial and temporal travel corridors); 2-behavioural studies (daily or seasonal activity patterns of individual animals); 3-home ranges of individual animals (quantitative estimates of area used in time and space); 4-habitat use and management; 5-survival studies (differentiate between emigration and mortality); 6-reproductive parameters (litter size, interbirth interval, adult survival and cub survival, and causes of mortality); 7-population size (using mark–recapture methods) and bio-telemetry, among others [3-7]. Such questions could not have been answered using other research approaches, such as land or aerial tracking, scat analysis, DNA analysis, detector dogs, or camera trapping [8-10].

Despite the pivotal advantages of the radio-collars, they were not applied, to the best of our knowledge, on wildlife disease monitoring.

*Sarcoptes scabiei* affects humans and a wide range of mammalian hosts worldwide [11,12]. It is an opportunistic parasite [13], incomprehensibly emerging and re-emerging with neglected navigating web, through which *S. scabiei* move from one to another host [14].

*S. scabiei* entails significant mortality in both wild and domestic animals, with considerable economic losses [15-17], and ravages in human populations [18].

Iberian ibex (*Capra pyrenaica*) population from Sierra Nevada mountain range in Spain is one of the most affected by *S. scabiei*[19-21]. Nonetheless, there are no clear studies about (i) the possible resistance of Iberian ibex to *S. scabiei*, (ii) the average survival time of the non-resistant animals, and (iii) the recovery time of the resistant ones, if any. Such aspects are almost impossible to approach by the direct observation of the affected animals, based on the limited access to different parts of Sierra Nevada because of the harsh climatology and territory of this mountain range and the absence of adequate roads. And hence the pivotal role of the gps-gsm radio collars, which allow localizing the marked animal at any time.

## Methods

### Animals capturing, radio collaring, monitoring and mange infection evaluation

**Figure 1:**
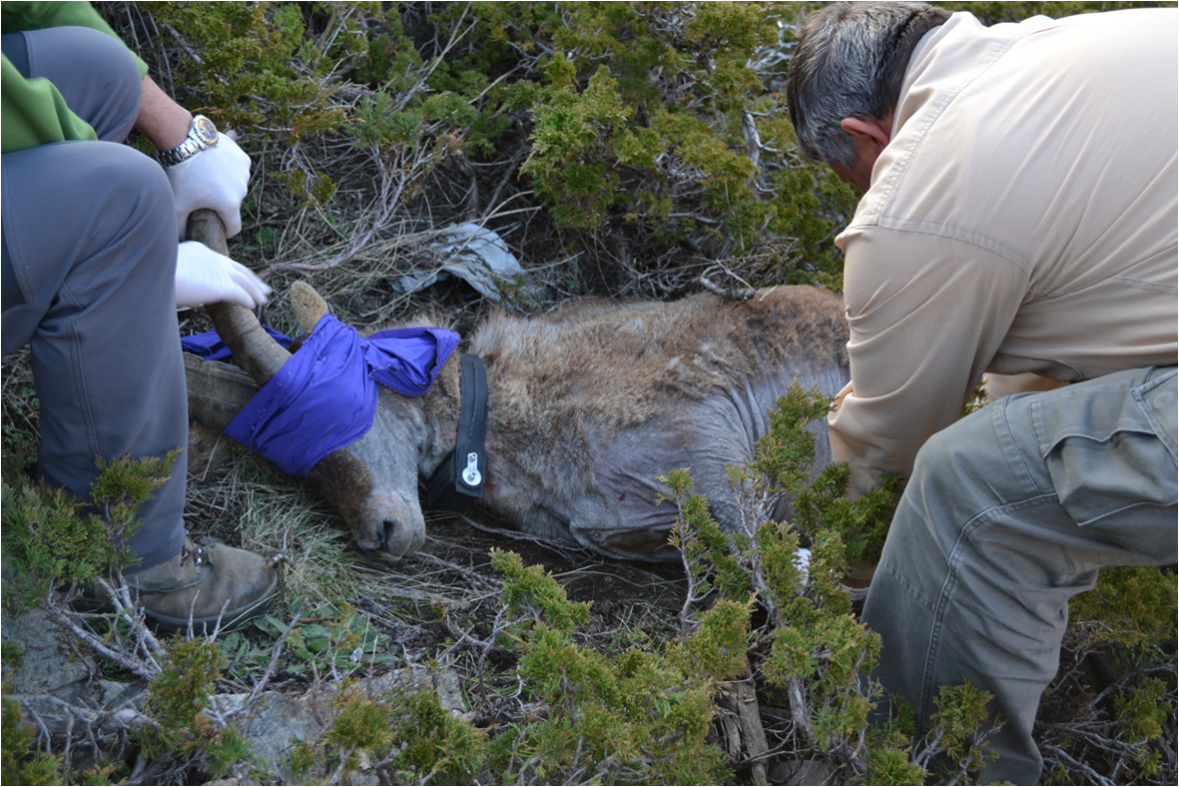
Mangy Iberian ibex capturing and gps-radio collaring, Sierra Nevada, Spain.

Between 06/02/2006 and 10/06/2013, 25 mangy Iberian ibex (ranged between 3-9 year old) were immobilized by darting using a mixture of xylazine (3 mg/Kg) and ketamine (3 mg/Kg). Iberian ibex were collared with gps + gsm collars (Microsensory, Córdoba, Spain, and Vectronic Aerospace, Germany) (Figure 1). The movement of each radio-collared animal was followed online using the webpage of the fabricant (http://www.wildgps.com). This webpage permits following the real-time movement of the animals, being updated every 3 hours. This allowed us easily localize the collared animals and hence the observation of the development of mange lesions.

The range estimated, once capturing the mangy Iberian ibex, visually (or by using binoculars or telescope when they observed the animal from far distance) the surface area of skin with sarcoptic lesions and animals were assigned to a mange field category on the basis of the area affected. The collared animals were moderately or slightly affected by mange, with less than 50% of the animal skin apparently affected. This category corresponds to less than 1324 ± 551 mite/cm^2^ of the affected animal skin [22].

### Ethics

This study complies the Spanish and the Andalusian laws regarding bioethics and animal welfare. Sierra Nevada National Park approved this study.

## Results and discussion

**Figure 2:**
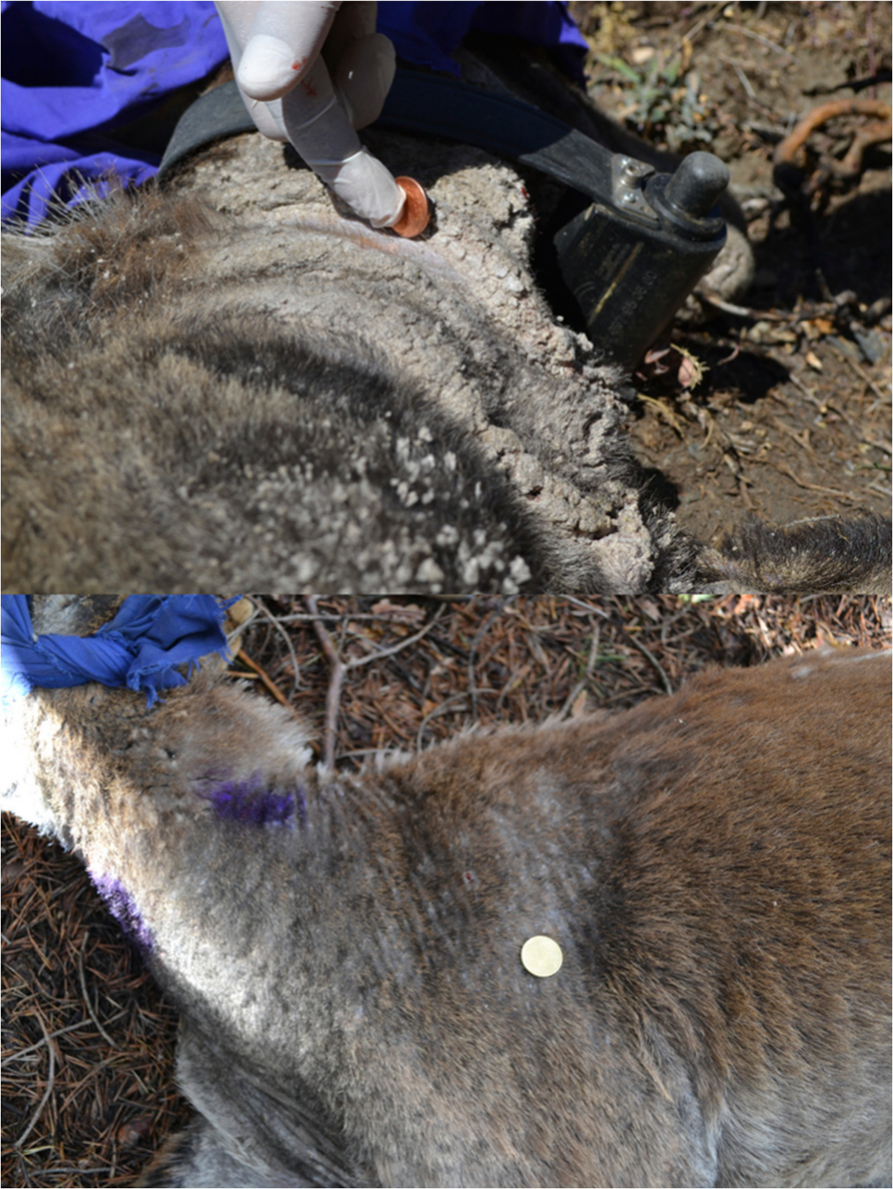
Photos showing the decrease of mange lesions and the recover of the normal skin and hair of one collared resistant Iberian ibex (up = before, down = after), Sierra Nevada, Spain.

**Figure 3:**
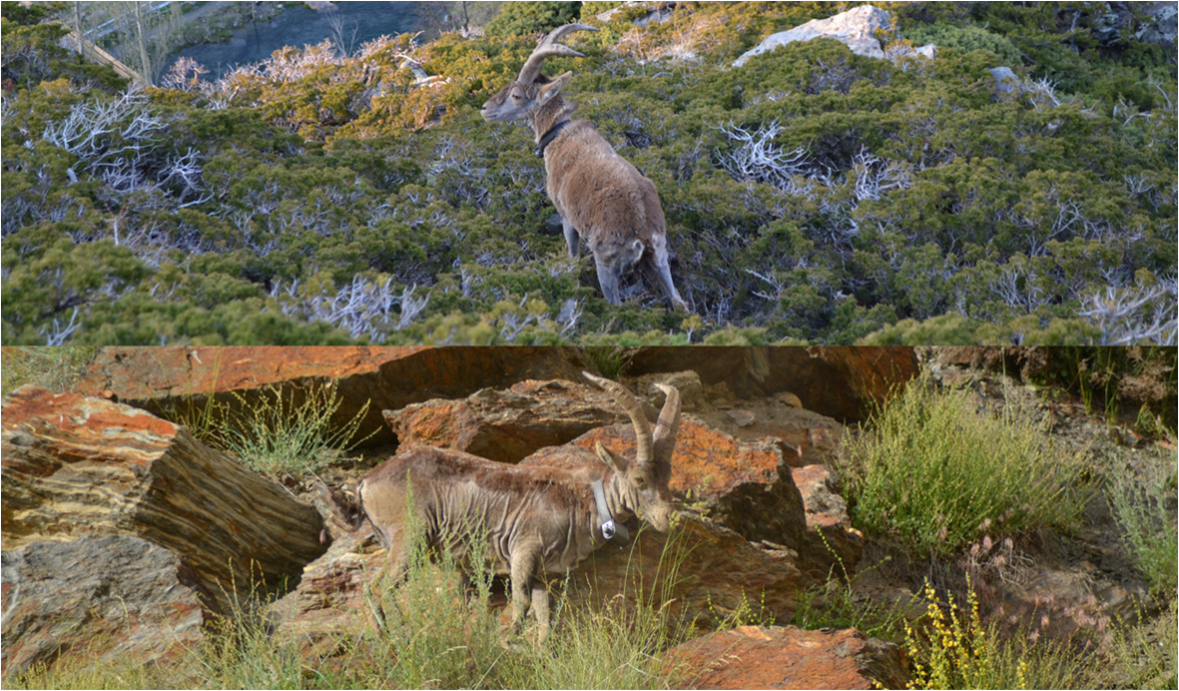
Photos showing the increase of mange lesions and the loss of hair of one collared non-resistant Iberian ibex (up = before, down = after), Sierra Nevada, Spain.

**Figure 4:**
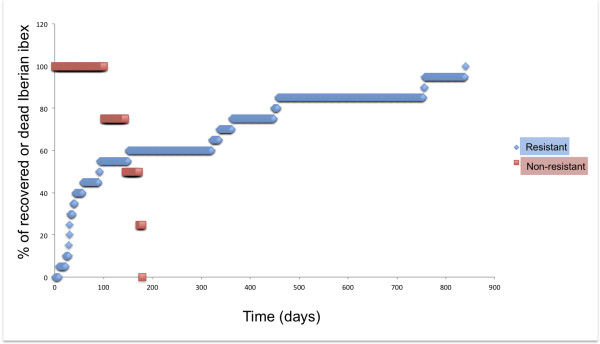
Recovery and survival time (days) of the resistant and non-resistant, respectively, mangy Iberian ibex from Sierra Nevada, Spain.

Mange infections in 20 (80% of the total) Iberian ibex were self-limited and the damaged skin surface decreased progressively, recovering new hair (Figure 2). The average time for total recovery was 245 ± 277 days. Meanwhile, other five (20% of the total) Iberian ibex showed significant increase of mange infections by the amplification of the damaged skin, until animal death (Figure 3). The average survival time was 121 ± 71 days, longer than that described by León-Vizcaíno *et al*. [23] in Cazorla, Segura and Las Villas Mountains. Non-resistant animals survived at least 100 days, while all of them were dead in less than 200 days. Sixty per cent of the resistant animals were recovered with less than 200 days (Figure 4).

This is the first use of radio- collars for wildlife diseases monitoring, which allowed us to detect the presence of resistant free-ranging Iberian ibex in Sierra Nevada, and to estimate the recovery time, together with the survival time of the non-resistant ones. These results, with the cautions regarding the low sampling size, are of pivotal interest for the future conservation and management of the Iberian ibex in Spain, and could be used as a model for the world monitoring and management of the affected wild animal species. These results support the policy of Sierra Nevada National Park authorities, who remove only the highly or severly mangy Iberian ibex (with more than 50% of the animal skin is apparently affected), but not the moderately or slightly affected (with less than 50% of the animal skin is apparently affected).

The radio- collars could be of great help for wildlife diseases treatment. In the case of *S. scabiei*, affected animals usually need 2-3 doses of drug (such as ivermectin) with a temporal interval of 2-4 weeks, to guarantee efficient treatment. However, this is impracticable with wild animals, due to climatological, territorial and logistical factors, especially with the elusive species [24]. And hence the radio-collars could be of great help, allowing localizing the animals under treatment whenever is necessary for repeated treatment purpose, or just to observe the efficacy and results of the previous treatment.

*Sarcoptes* is mainly transmitted by host-to-host direct contact, and hence better understanding of the interaction between affected and not-affected individuals is of pivotal interest to clarify the epidemiology of this disease [14], which could be achieved by using radio collars.

## Conclusions

In the present study we report, for the first time, the successful use of radio collars for wildlife diseases monitoring using Iberian ibex/*S. scabiei* as a model. By using radio collars we documented that most of the *Sarcoptes*-infected Iberian ibex are resistant to this disease, and we estimated the average time for Iberian ibex recovering from mange infection and the average survival time of the non-resistant ones. We expect wider use of radio-collars for wild animals diseases monitoring, affected/not-affected animals interaction, and treatment efficacy, among others.

## Competing interests

The authors declared that they have no competing interests.

## Authors’ contributions

JEG, PF, RCS, SA and JMP conceived and designed the experiments. JEG, PF, FJCM, RCS and JMP performed the fieldwork experiments. Manuscript was analysed, discussed and written by all co-authors. All authors read and approved the final version of the manuscript.
